# A dynamic covalent polymeric antimicrobial for conquering drug‐resistant bacterial infection

**DOI:** 10.1002/EXP.20210145

**Published:** 2022-05-23

**Authors:** Fan Huang, Xiaoyao Cai, Xiaoxue Hou, Yumin Zhang, Jinjian Liu, Lijun Yang, Yong Liu, Jianfeng Liu

**Affiliations:** ^1^ Key Laboratory of Radiopharmacokinetics for Innovative Drugs Chinese Academy of Medical Sciences, and Institute of Radiation Medicine Chinese Academy of Medical Sciences & Peking Union Medical College Tianjin P. R. China; ^2^ Engineering Research Center of Clinical Functional Materials and Diagnosis & Treatment Devices of Zhejiang Province Wenzhou Institute University of Chinese Academy of Sciences, Oujiang Laboratory (Zhejiang Lab for Regenerative Medicine, Vision and Brain Health) Wenzhou Zhejiang P. R. China

**Keywords:** antimicrobials, combination therapy, micelles, multidrug resistance, phenylboronic acid

## Abstract

Increasing bacterial drug resistance to antibiotics has posed a major threat to contemporary public health, which resulted in a large number of people suffering from serious infections and ending up dying without any effective therapies every year. Here, a dynamic covalent polymeric antimicrobial, based on phenylboronic acid (PBA)‐installed micellar nanocarriers incorporating clinical vancomycin and curcumin, is developed to overcome drug‐resistant bacterial infections. The formation of this antimicrobial is facilitated by reversible dynamic covalent interactions between PBA moieties in polymeric micelles and diols in vancomycin, which impart favorable stability in blood circulation and excellent acid‐responsiveness in the infection microenvironment. Moreover, the structurally similar aromatic vancomycin and curcumin molecules can afford π–π stacking interaction to realize simultaneous delivery and release of payloads. In comparison with monotherapy, this dynamic covalent polymeric antimicrobial demonstrated more significant eradication of drug‐resistant bacteria in vitro and in vivo due to the synergism of the two drugs. Furthermore, the achieved combination therapy shows satisfied biocompatibility without unwanted toxicity. Considering various antibiotics contain diol and aromatic structures, this simple and robust strategy can become a universal platform to combat the ever‐threatening drug‐resistant infectious diseases.

## Graphical abstract

A dynamic covalent polymeric antimicrobial, based on phenylboronic acid‐installed micellar nanocarriers incorporating vancomycin and curcumin, was developed to overcome drug‐resistant bacterial infections. Compared with monotherapy, this antimicrobial displayed more significant eradication of drug‐resistant bacteria in vitro and in vivo. This work could become a universal platform to combat the ever‐threatening drug‐resistant infectious diseases.

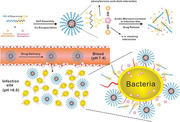

## 1 INTRODUCTION

The emergency of multidrug‐resistant (MDR) bacteria has become a public health concern, not only because of increased morbidity and mortality, but in terms of soaring treatment expenditure.^[^ ^1^ ^]^ Among a wide variety of MDR bacteria, methicillin‐resistant *Staphylococcus aureus* (MRSA), characterized by complete resistance to all other β‐lactam and cephalosporin antibiotics, is described as one of the toughest clinical problems.^[^ ^2^ ^]^ To date, vancomycin, a glycopeptide antibiotic, is employed as a first‐line drug and the last resort to treat MRSA‐induced diseases such as endocarditis, septicemia, and osteomyelitis.^[^ ^3^ ^]^ However, long‐term and frequent application of vancomycin put selective antibiotic pressure on the bacteria, resulting in the appearance of vancomycin‐resistant *S. aureus* over the past three decades.^[^ ^4^ ^]^ Rising vancomycin resistance has reportedly contributed to treatment failures and left therapy of staphylococcal infection a worldwide challenge.^[^ ^5^ ^]^ Therefore, there is an urgent need for the exploration of innovative and efficient strategies against *S. aureus* with vancomycin resistance.

At present, combination drug therapy has attracted a great amount of attention in the war against bacterial infections. Extensive researches reported that combination treatment of antimicrobial agents can exert synergism by means of acting on various targets or different pathways in bacteria.^[^ ^6^ ^]^ This approach possessed several benefits such as overcoming multidrug resistance, boosting drug efficacy, and reducing side‐effects.^[^ ^7^ ^]^ Nowadays vancomycin is often administered with other kinds of antibiotics, such as rifampin and gentamicin to broaden the antibacterial spectrum, whereas these regimens may induce some adverse effects, such as hepatotoxicity and nephrotoxicity.^[^ ^8^ ^]^ Cumulative evidence demonstrated that curcumin is a natural antibacterial compound extracted from turmeric with low toxicity.^[^ ^9^ ^]^ Curcumin has been proved to possess broad bactericidal activity against not only gram‐positive but also gram‐negative bacteria via interacting with cellular components of bacteria, such as cell membrane, protein and DNA, and inhibiting biofilm formation.^[^ ^10^ ^]^ Furthermore, it has been found to exhibit synergistic effects in combination with various antimicrobials, including β‐lactams, cephalosporins, glycopeptides, aminoglycosides, and fluoroquinolones.^[^ ^11^ ^]^ More importantly, recent studies showed that curcumin has the capability to enhance the antimicrobial efficacy of vancomycin significantly,^[^ ^12^ ^]^ suggesting the potential for reversing the resistance to vancomycin. Nevertheless, the combination treatment of curcumin and vancomycin is severely restricted from applying in vivo by distinct physicochemical properties. Curcumin is a hydrophobic compound with low bioavailability and rapid metabolism,^[^ ^13^ ^]^ while vancomycin is a hydrophilic drug that exhibits 10% to 50% ratio of protein binding and wide distribution.^[^ ^14^ ^]^ Different water solubility and pharmacokinetics exhibited by curcumin and vancomycin resulted in inconsistent biodistribution and independent elimination after injection, which would consequently induce a poor synergistic antibacterial effect in vivo.^[^ ^15^ ^]^ Currently, nanocarriers are extensively used to deliver a wide range of drugs for combination treatment, satisfying diverse therapeutic needs.^[^ ^16^ ^]^ Previous researches showed that nanocarriers are able to achieve co‐delivery of hydrophobic and hydrophilic drugs to increase bioavailability, improve stability, and enhance therapeutic effects.^[^ ^17^ ^]^ More importantly, nanocarriers can be manufactured to possess stimuli‐responsive properties such as pH‐responsiveness, thermo‐responsiveness, and redox‐sensitivity, leading to the fact that nanocarriers are able to respond specifically to the unique pathological alteration at the target site.^[^ ^18^ ^]^ However, drug encapsulation by the majority of the traditional nanocarriers is mostly driven by hydrophilic/hydrophobic interaction or electrostatic effect, which still leads to unsatisfactory drug loading and undesired drug release. Thus, it is highly demanded to introduce suitable intermolecular interactions to realize a favorable combination of vancomycin and curcumin within nanocarriers for conquering vancomycin resistance.

**SCHEME 1:**
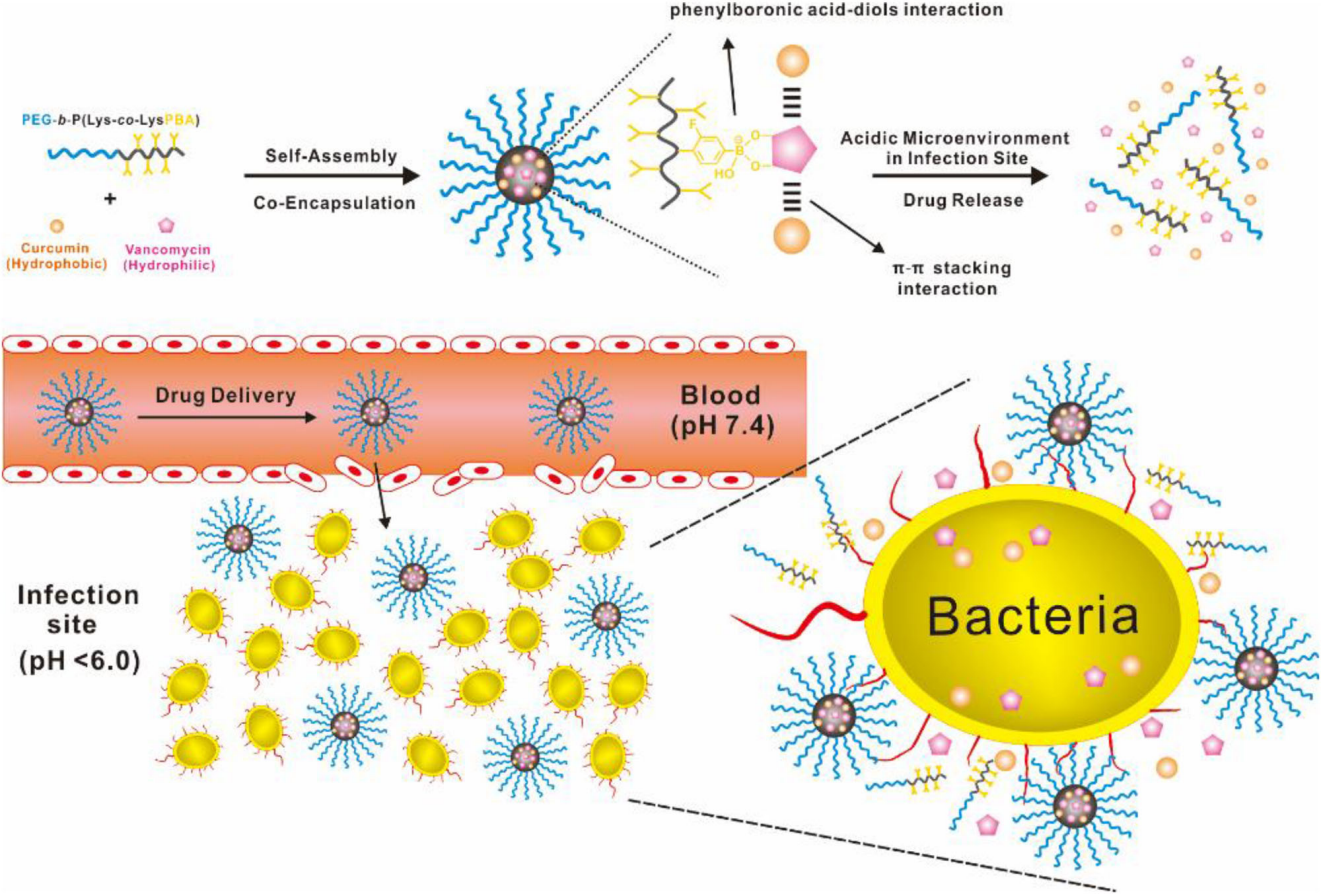
Schematic illustration of self‐assembly of dynamic covalent polymeric antimicrobials and their mechanism to combat drug‐resistant bacterial infection

Herein, we reported a dynamic covalent polymeric antimicrobial by installing PBA moieties into the nanocarriers and co‐encapsulating vancomycin and curcumin for conquering vancomycin‐resistant *S. aureus* infections (Scheme 1). This antimicrobial was first constructed through PBA‐diol interaction between PBA‐bearing amphiphilic copolymer, poly(ethylene glycol)‐*block*‐poly(lysine‐*co*‐lysine‐phenylboronic acid) (PEG‐*b*‐P(Lys‐*co*‐LysPBA)), and glycopeptide antibiotic vancomycin. With multiple‐ringed structures in the vancomycin, aromatic polyphenol drug curcumin could be effectively encapsulated in the core of the above vancomycin‐packaged nanocarrier through π–π stacking interaction. In this strategy, multiple interactions in the core of as‐prepared polymeric antimicrobials endowed them with satisfying formulation stability and two drug loading capacity. More importantly, the boronate ester bond formed by PBA groups in the copolymer and vicinal diols of vancomycin was a well‐known pH‐sensitive dynamic covalent bond, which could be cleaved under the acidic microenvironment of the infection site and awarded the antimicrobials with an efficient and simultaneous release of two payloads to exert a decent synergistic effect. Furthermore, we found that such dynamic covalent polymeric antimicrobial could significantly eradicate vancomycin‐resistant *S. aureus* infection in a murine model without any systemic toxicity. Consequently, this work represented an efficient and safe strategy to fight against antibiotic resistance.

## 2 RESULTS AND DISCUSSION

### 2.1 Preparation and characterization of dynamic covalent polymeric antimicrobials

**FIGURE 1:**
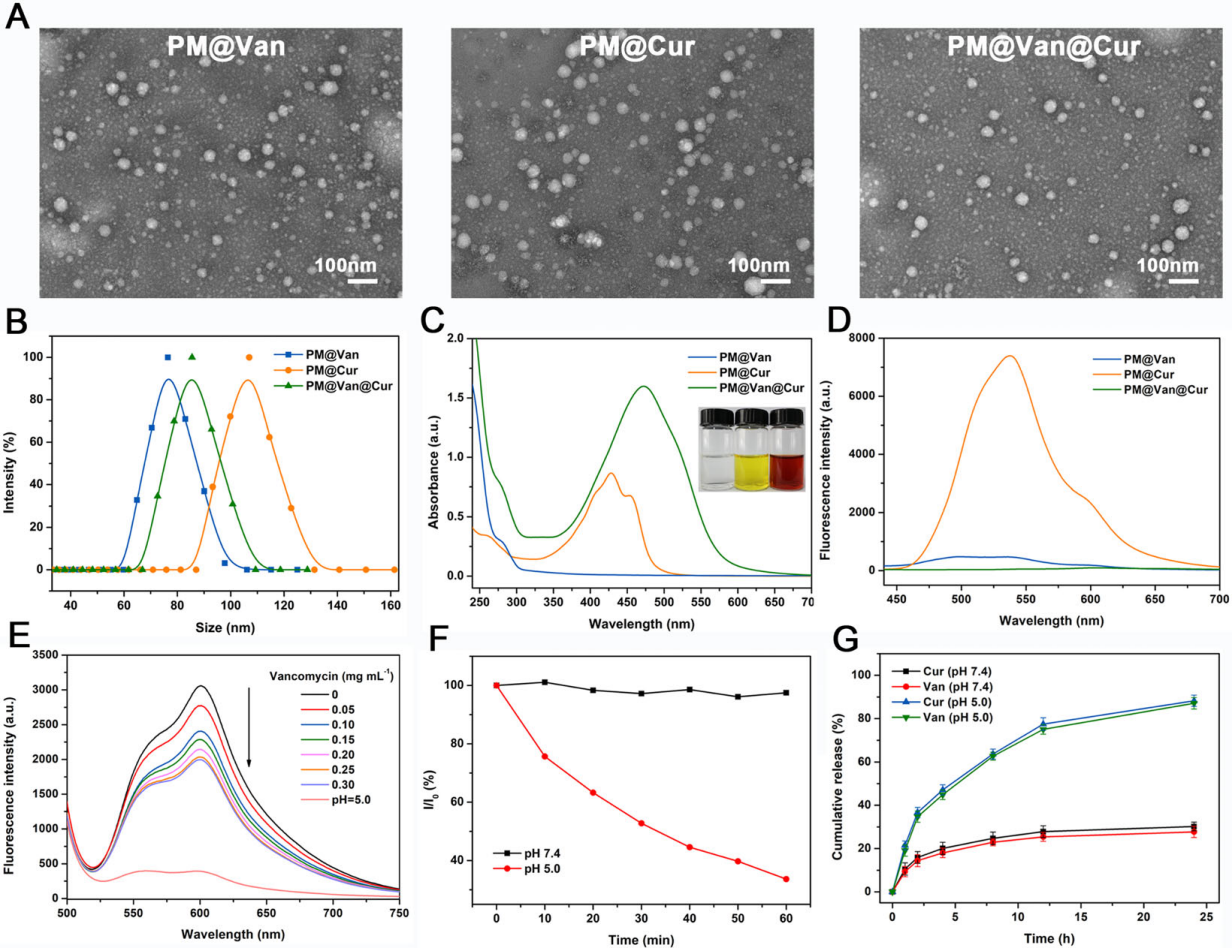
Characterization of dynamic covalent polymeric antimicrobials. (A) TEM images, (B) size distributions, (C) UV–vis absorption spectra, and (D) fluorescence spectra of different PM@Van, PM@Cur, and PM@Van@Cur. (E) Fluorescence spectra of mixture solution consisting of ARS and PEG‐*b*‐P(Lys‐*co*‐LysPBA) under different amounts of vancomycin titration and pH 5.0. (F) Relative light scattering intensity variation of the PM@Van@Cur solutions at different pH values. (G) In vitro release profiles of vancomycin and curcumin from PM@Van@Cur at different pH conditions

In this study, the dynamic covalent polymeric antimicrobials (named as PM@Van@Cur) were prepared in a one pot process due to the straightforward self‐assembly of amphiphilic copolymer PEG‐*b*‐P(Lys‐*co*‐LysPBA) with vancomycin and curcumin. Meanwhile, the polymeric micelles only packaging vancomycin (named as PM@Van) or curcumin (named as PM@Cur) acted as the controls. As presented in Figure 1A, the transmission electron microscopy images showed that the above three micelles were all well dispersed with spherical structures. Their average hydrodynamic diameter was approximately 75, 85, and 110 nm, respectively, as determined by dynamic light scattering (Figure 1B). The slightly increased size of PM@Van@Cur than PM@Van was because of co‐encapsulation of two drugs, while the larger size of PM@Cur than PM@Van@Cur could be attributed to the fact that the hydrophobic interaction as a major driving force for drug encapsulation was weaker than that of the dynamic covalent bonds.^[^ ^19^ ^]^ The UV–vis absorption spectra results (Figure 1C) showed that PM@Van and PM@Cur exhibited characteristic peaks of vancomycin (around 280 nm) and curcumin (around 425 nm), which indicated that these micelles successfully entrapped vancomycin and curcumin, respectively. Whereas in the case of PM@Van@Cur, it not only showed a superposition spectrum of PM@Van and PM@Cur, but also displayed a pronounced redshift and absorption enhancement at the same concentration, confirming the existence of π–π stacking interaction and successful co‐encapsulation of vancomycin and curcumin.^[^ ^20^ ^]^ Moreover, the inset photograph showed that these micelles possessed different appearances and colors without any aggregation, which further suggests the satisfactory preparation of micelles with different payloads and good dispersibility. The fluorescence spectra of these three micelles are shown in Figure 1D. It could be observed that PM@Cur expressed a broad emission band with a maximum peak at about 530 nm with an excitation wavelength of 420 nm, while remarkable fluorescence quenching occurred in the case of PM@Van@Cur due to the π–π stacking interaction between vancomycin and curcumin.^[^ ^21^ ^]^

To further confirm the PBA‐diols interaction in the micelles, commercially available alizarin red S (ARS) was used as a fluorescence probe to monitor the formation of the phenylboronate ester.^[^ ^22^ ^]^ As shown in Figure 1E, the fluorescence intensity of ARS at around 600 nm remarkably increased on incubation with PEG‐*b*‐P(Lys‐*co*‐LysPBA), suggesting the binding of ARS with PBA groups in copolymer. Upon the addition of vancomycin, the fluorescence intensity gradually decreased with the increasing vancomycin concentration, which was because of the competitive binding of vancomycin to PBA groups to replace the ARS, leading to the formation of phenylboronate ester between vancomycin and PEG‐*b*‐P(Lys‐*co*‐LysPBA). Moreover, the fluorescence intensity further dramatically declined after adjusting the pH to 5.0, indicating that the binding of vancomycin to PBA groups in micelles could be broken under an acidic environment.

Then, the in vitro stability and pH‐responsiveness of PM@Van@Cur were investigated by monitoring the change of relative light scattering intensity (*I*/*I*_0_) as a function of time. As shown in Figure 1F, the *I*/*I*_0_ of the micelle solution was almost constant at pH 7.4, implying the good stability of PM@Van@Cur under a physiologically neutral condition. When the pH of the micelle solution was adjusted to 5.0, the *I*/*I*_0_ of PM@Van@Cur decreased continuously during the measurement, suggesting the disintegration of micelles due to the breakage of the phenylboronate ester under the acidic environment.

Since the infection sites always displayed acidic microenvironment with low pH levels, the in vitro release profiles of PM@Van@Cur were examined under different pH conditions. The drug loading capacity (DLC) of PM@Van@Cur for vancomycin was as high as 21.4% (Table S1), which was much higher than that of conventional polymeric micelles (typically lower than 10%).^[^ ^23^ ^]^ Due to the fact that the control PM@Cur was prepared through hydrophobic interaction between PLys(Z) and curcumin and its DLC for curcumin was around 5.1%, we regulated the feed amount of the drug to guarantee the same DLC of PM@Van@Cur for curcumin. As shown in Figure 1G, the cumulative release of vancomycin and curcumin was less than 30% in 24 h under the physiological pH of 7.4. In contrast, the drug release rate became evidently faster and the cumulative releases were up to nearly 90% at pH 5.0. These results revealed that the phenylboronate ester bond in PM@Van@Cur endowed them with the commendable ability of pH‐triggered release in infection sites. Moreover, the release trend of vancomycin and curcumin were similar no matter at pH 7.4 or 5.0, indicating that PM@Van@Cur offered a good possibility to produce a synergistic effect of drugs.

### 2.2 In vitro antibacterial activity evaluation of dynamic covalent polymeric antimicrobials

**FIGURE 2:**
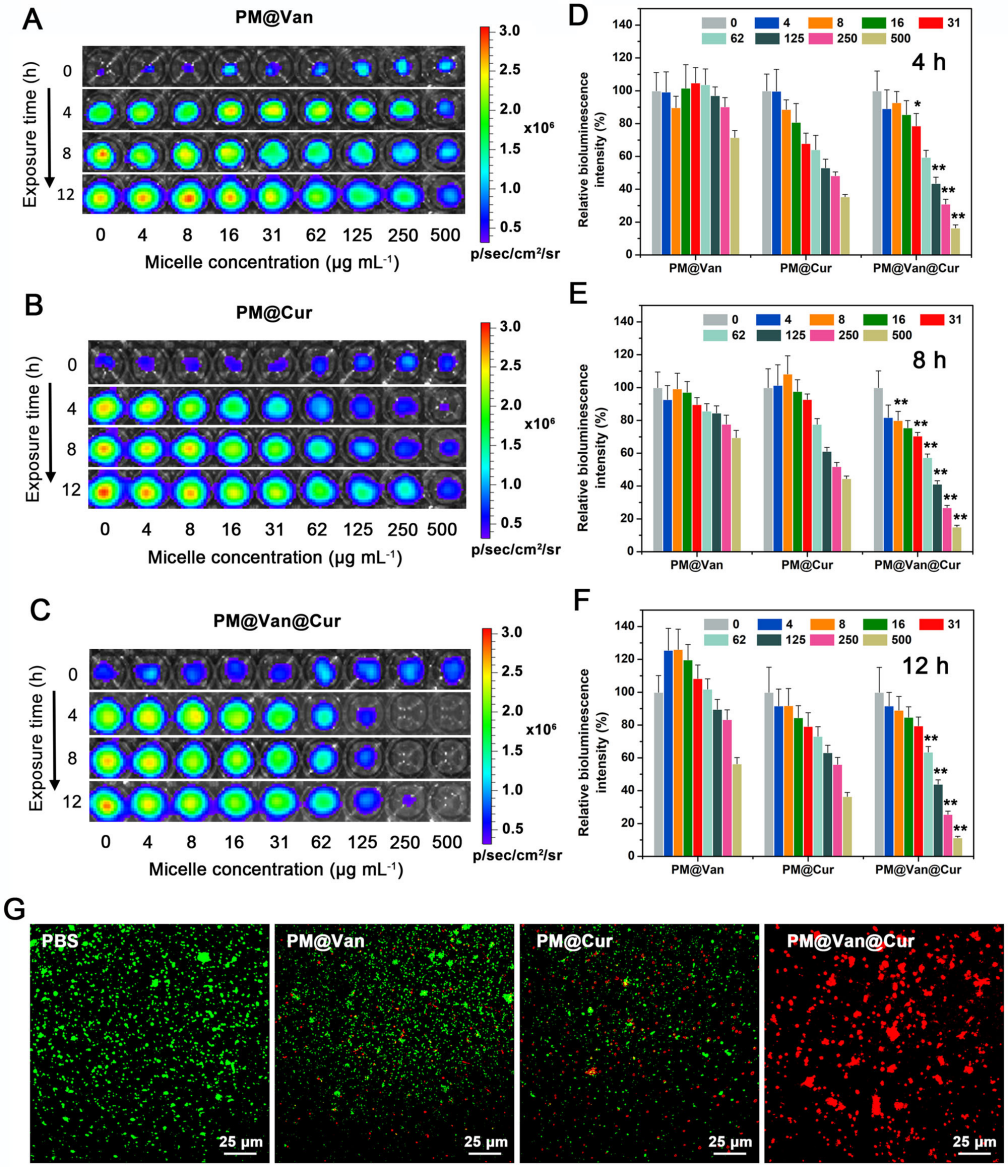
In vitro antibacterial activity of dynamic covalent polymeric antimicrobials. Bioluminescence images of *Staphylococcus aureus* Xen 36 after exposure to different concentrations of (A) PM@Van, (B) PM@Cur, and (C) PM@Van@Cur for different times. Relative bioluminescence intensities of *S. aureus* Xen36 after being treated with different micelles for (D) 4 h, (E) 8 h, and (F) 12 h. Bioluminescence of the bacteria exposed to PBS is 100%. All data are expressed as means ± SD over sextuplicate experiments. *p*‐Values were calculated by one‐way ANOVA. *, ** indicated *p* < 0.05, *p* < 0.01 different from all other treatments at the same concentration. (G) Fluorescence images of *S. aureus* Xen36 after incubation with PBS, PM@Van, PM@Cur, and PM@Van@Cur using live/dead staining assays

To assess the antibacterial activity of dynamic covalent polymeric antimicrobials, vancomycin‐resistant *S. aureus* Xen36 with bioluminescence property was used as the bacteria model.^[^ ^24^ ^]^ We incubated the *S. aureus* Xen36 with PM@Van, PM@Cur, and PM@Van@Cur for different times (4, 8, and 12 h), and then took the bioluminescence images in which the bioluminescent intensity was negatively correlated with the antibacterial effect. Figure 2A,B shows that PM@Van and PM@Cur display very weak bacterial killing activity because the strong bioluminescent signals could be obviously detected even at the highest micelle concentration. However, the bioluminescent intensity of *S. aureus* Xen36 obviously diminished with the increasing concentration of PM@Van@Cur, and there was almost no bioluminescent signal when the concentrations reached more than 250 μg ml^–1^ (Figure 2C), suggesting that the antibacterial activity was significantly enhanced upon the combination of vancomycin and curcumin. The quantitative analysis of these bioluminescent data is shown in Figure 2D–F, which also supports the above observations. The live/dead bacterial cell viability assay was performed to further evaluate the antibacterial effect of the above three micelles. In this experiment, the live bacteria were dyed by green fluorescent probe Calcein‐AM while the dead ones could be labeled with propidium iodide (PI) emitting red fluorescence. As shown in Figure 2G, no detectable red fluorescence could be seen in the PBS group, symbolizing that the bacteria without micelle treatment were nearly all alive after incubation at 37°C. When the PM@Van and PM@Cur were applied, only a few red fluorescence was observed, which indicated that these two micelles merely exhibited weak toxicity to the vancomycin‐resistant *S. aureus* Xen36. In contrast, the bacteria treated with PM@Van@Cur showed strong red fluorescence and no green fluorescence signal could be seen, manifesting that almost all the bacteria were dead. All the above results proved that PM@Van@Cur could effectively overcome the drug resistance of *S. aureus* Xen36 and kill them through a synergic antibacterial effect.

To further confirm the synergism between vancomycin and curcumin against *S. aureus* Xen36, the checkerboard assay was carried out. As shown in Figure S1, the minimal inhibitory concentration (MIC) of vancomycin and curcumin for *S. aureus* Xen36 was 32 and 500 μg ml^–1^ separately. When vancomycin was combined with curcumin, its MIC was dropped by 16 times, from 32 to 2 μg ml^–1^. In terms of fractional inhibitory concentration index (Table S2), it was calculated to be 0.093 < 0.5, indicative of an excellent synergistic effect between vancomycin and curcumin.

### 2.3 Eradication of vancomycin‐resistant staphylococcal infection in vivo

Encouraged by the above results in vitro, we subsequently investigated the eradication effect of *S. aureus* Xen36 infection in a murine model. The murine infected model was established through subcutaneous inoculation of *S. aureus* Xen 36 in the right flank of mice. Two days after bacteria inoculation, the bioluminescence signals of infection sites in mice were detected to ensure the successful establishment of the model. To evaluate the accumulation of the polymeric antimicrobial at the site of the infection, in vivo fluorescence imaging assay was carried out. Fluorescence probe Cy5 structurally similar to curcumin was used to label polymeric antimicrobial which was named as PM@Van@Cy5. Then we injected it intravenously into *S. aureus* Xen 36‐bearing mice. Free Cy5 was performed as a control. As shown in Figure S2, fluorescence of free Cy5 was dim at 0.5 h and then disappeared 1 h after infection, indicating the rapid clearance of Cy5 in the blood circulation. In contrast, the fluorescence of PM@Van@Cy5 could be observed clearly in the infected region and reached the maximum at 4 h, after which it dropped gradually and still existed at 24 h. The quantitative analysis in Figure S3 was consistent with the above results. Collectively, the abovementioned results demonstrated the successful delivery and retention of vancomycin and curcumin at the site of infection.

**FIGURE 3:**
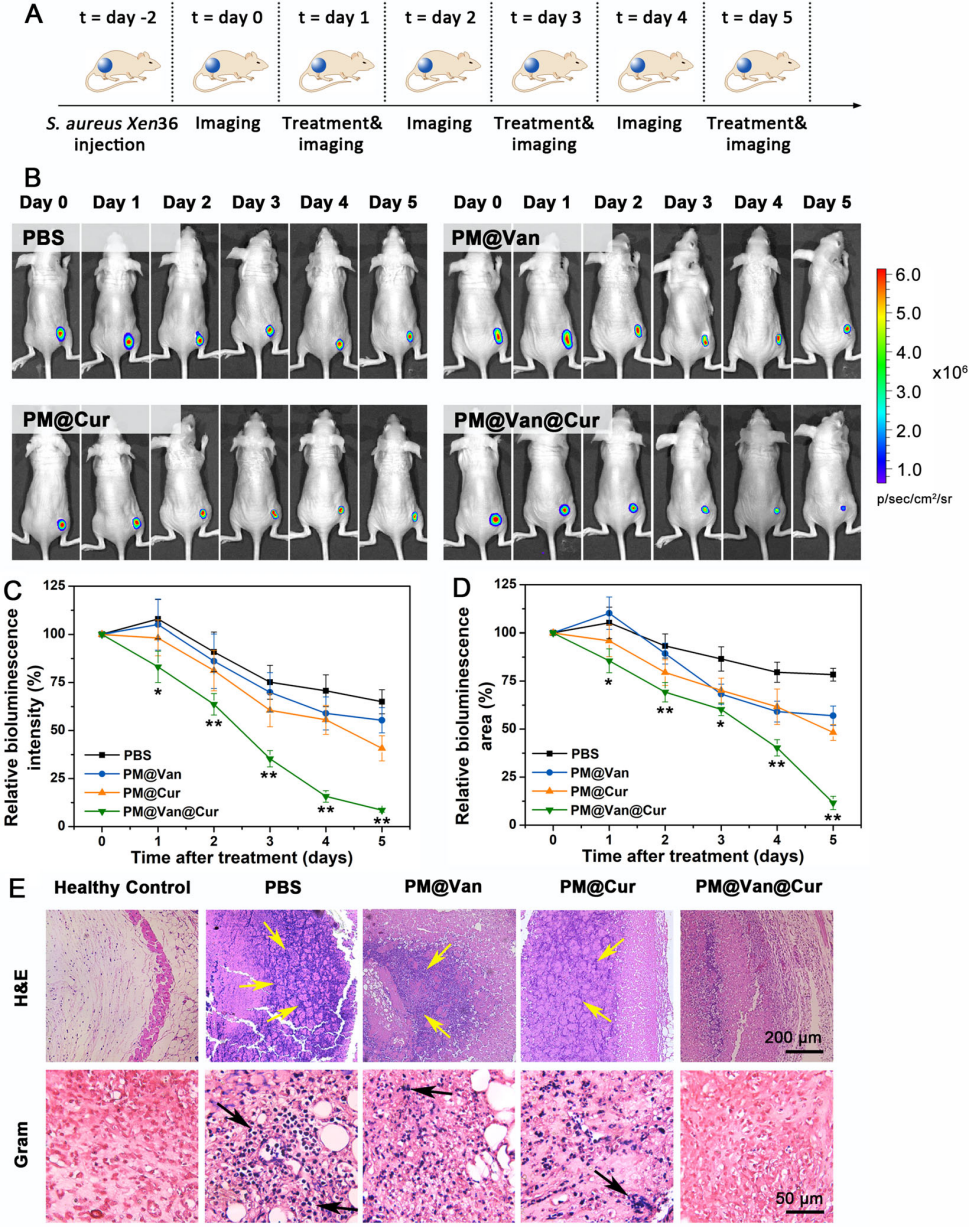
Eradication effect of dynamic covalent polymeric antimicrobials for vancomycin‐resistant staphylococcal infection in vivo. (A) Workflow for *Staphylococcus aureus* Xen 36 infection, treatment, and bio‐optical imaging in mice. (B) Time series of bioluminescence images for *S. aureus* Xen36‐infected mice after treatment with PBS, PM@Van, PM@Cur, and PM@Van@Cur. (C) Relative bioluminescence intensity and (D) relative bioluminescent area arising from the infection site as a function of time after different treatments. Bioluminescence intensity of day 0 was set at 100%. Error bars represent SD values over six mice per group. *p*‐Values were calculated by one‐way ANOVA. *, ** indicate *p* < 0.05, *p* < 0.01 different from all other treatments. (E) Micrographs of H&E and Gram‐stained tissues of infected mice after different treatments. The untreated healthy mice were used as healthy control. Yellow arrows indicate the presence of inflammatory cells. Black arrows indicate the presence of *S. aureus*

For in vivo antibacterial assay, *S. aureus* Xen 36‐bearing mice were treated with respective agents, and the therapeutic effect was monitored by imaging, according to the workflow in Figure 3A. The time series of bioluminescent images are presented in Figure 3B and the relative bioluminescence intensity and infected area are shown in Figure 3C,D. The mice injected with PM@Van showed little difference with the PBS group in terms of bioluminescent intensity and area, further indicating the vancomycin resistance of *S. aureus* Xen36 and failure of treatment of PM@Van. The PM@Cur group displayed a faster decrease of bacterial bioluminescence compared with the PM@Van group, but there was still about 50% infection after treatment at 5 days. However, the PM@Van@Cur induced the fastest clearance of the infection and to the lowest levels (<10%) among all the groups. Moreover, histological images after H&E staining and Gram staining are shown in Figure 3E. Compared with the healthy control, there were lots of inflammatory cells infiltrating and gathering (as yellow arrows indicated) in the H&E‐stained tissue sections of PBS group due to the bacterial infection. The PM@Van and PM@Cur groups showed only a little lightened inflammation, while the inflammatory cells decreased remarkably after treatment with PM@Van@Cur. For the micrographs of Gram‐stained tissues, large numbers of staphylococci (as black arrows indicated) were observed in the PBS group and the treatment after PM@Van and PM@Cur could not effectively eliminate these bacteria. Nevertheless, almost no bacteria could be observed in the tissue sample of PM@Van@Cur treatment group. Therefore, these results demonstrated that the PM@Van@Cur possessed an excellent eradicating effect for vancomycin‐resistant staphylococcal infection in vivo.

**FIGURE 4:**
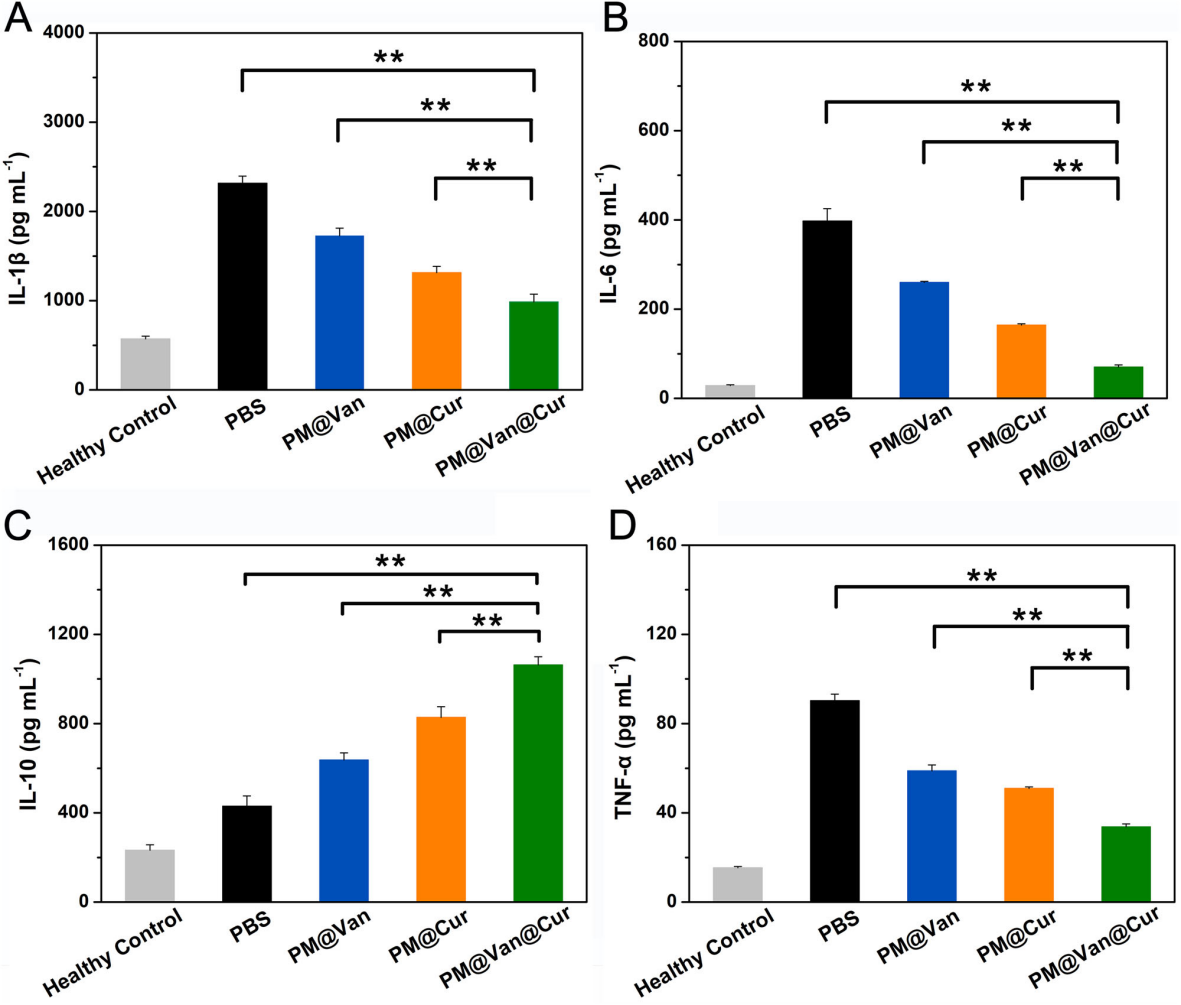
Quantitative analysis of (A) IL‐1β, (B) IL‐6, (C) IL‐10, and (D) TNF‐α of vancomycin‐resistant staphylococcal infection mice after treatment with PBS, PM@Van, PM@Cur, and PM@Van@Cur. The untreated healthy mice were used as healthy control. The data are presented as mean ± SD (*n* = 6). *p*‐Values were calculated by one‐way ANOVA, *, ** indicated *p* < 0.05, *p* < 0.01 different from other treatments

Since numerous pro‐inflammatory cytokines such as IL‐1β, IL‐6, and TNF‐α and anti‐inflammatory IL‐10 play an important role in the progression of bacterial infection disease, the capability of different treatment groups to regulate these cytokines was therefore studied.^[^ ^25^ ^]^ As shown in Figure 4, the PM@Van@Cur could significantly decrease the levels of IL‐1β, IL‐6, and TNF‐α and increase the amount of IL‐10 compared with any other treatment groups, signifying that our combination therapy based on dynamic covalent polymeric antimicrobials had superior inflammatory cytokine regulation ability to the other mono‐therapy, contributing to alleviate the infection‐induced inflammation.

### 2.4 Antibacterial mechanism of dynamic covalent polymeric antimicrobials

As mentioned above, PM@Van@Cur displayed a remarkable synergistic killing effect on *S. aureus* Xen36 in vitro and in vivo. Previous studies have proved that curcumin could disrupt the bacterial membrane, leading to permeabilization and cell death.^[^ ^26^ ^]^ Meanwhile, vancomycin has been proved to exert a bactericidal effect mainly via damaging the cell wall, thereby triggering cell rupturing.^[^ ^27^ ^]^ Hence, we presumed that the combination treatment of vancomycin and curcumin could kill *S. aureus* by destroying bacterial cell membrane structure collaboratively. Protein leakage assay was thus conducted. Figure S4 shows that three kinds of treatments could all induce intracellular protein leaking. However, compared with vancomycin alone or curcumin alone, there was a distinct increase in leakage of intercellular protein for the combination of vancomycin and curcumin, which indicated the combination therapy had a greater influence on membrane integrity than monotherapy. Thereby, PM@Van@Cur could exert bactericidal effects by damaging the bacterial membrane synergistically.

### 2.5 Biosafety assessment of dynamic covalent polymeric antimicrobials

**FIGURE 5:**
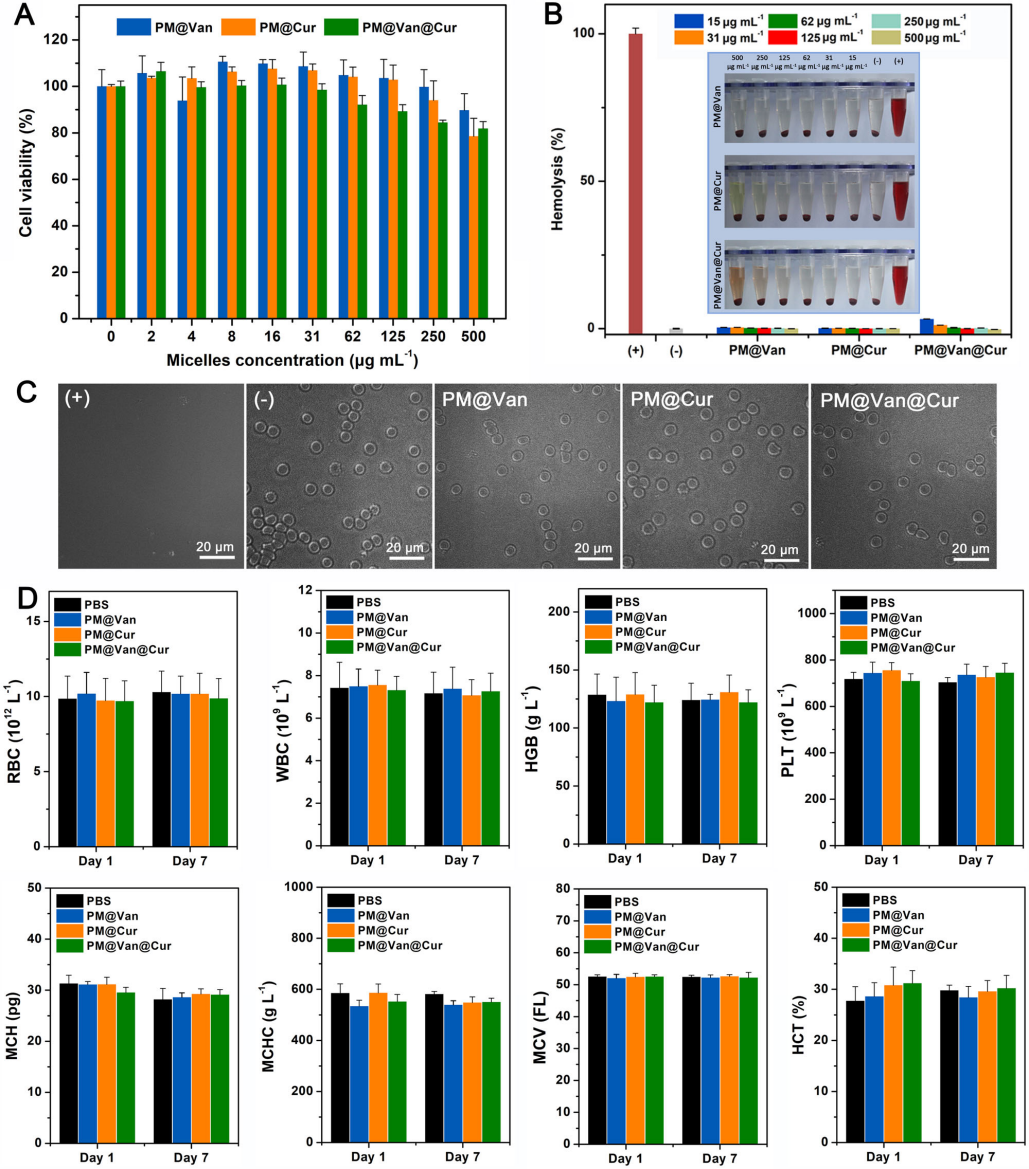
Biocompatibility of dynamic covalent polymeric antimicrobials. (A) Cell cytotoxicity of NIH3T3 cells after treatment with PM@Van, PM@Cur, and PM@Van@Cur. (B) Hemolysis analysis of PM@Van, PM@Cur, and PM@Van@Cur at different concentrations. Erythrocytes incubated with PBS and 0.1% (by volume) Triton X‐100 in PBS buffer were served as negative (−) and positive (+) controls, respectively. (C) Micrograph of erythrocytes harvested after hemolysis assay with different groups. (D) Major blood parameters of mice treated with PBS, PM@Van, PM@Cur, and PM@Van@Cur. Blood samples were taken on days 1 and 7 after the corresponding treatment. Error bars denote SD over six mice in each group

**FIGURE 6:**
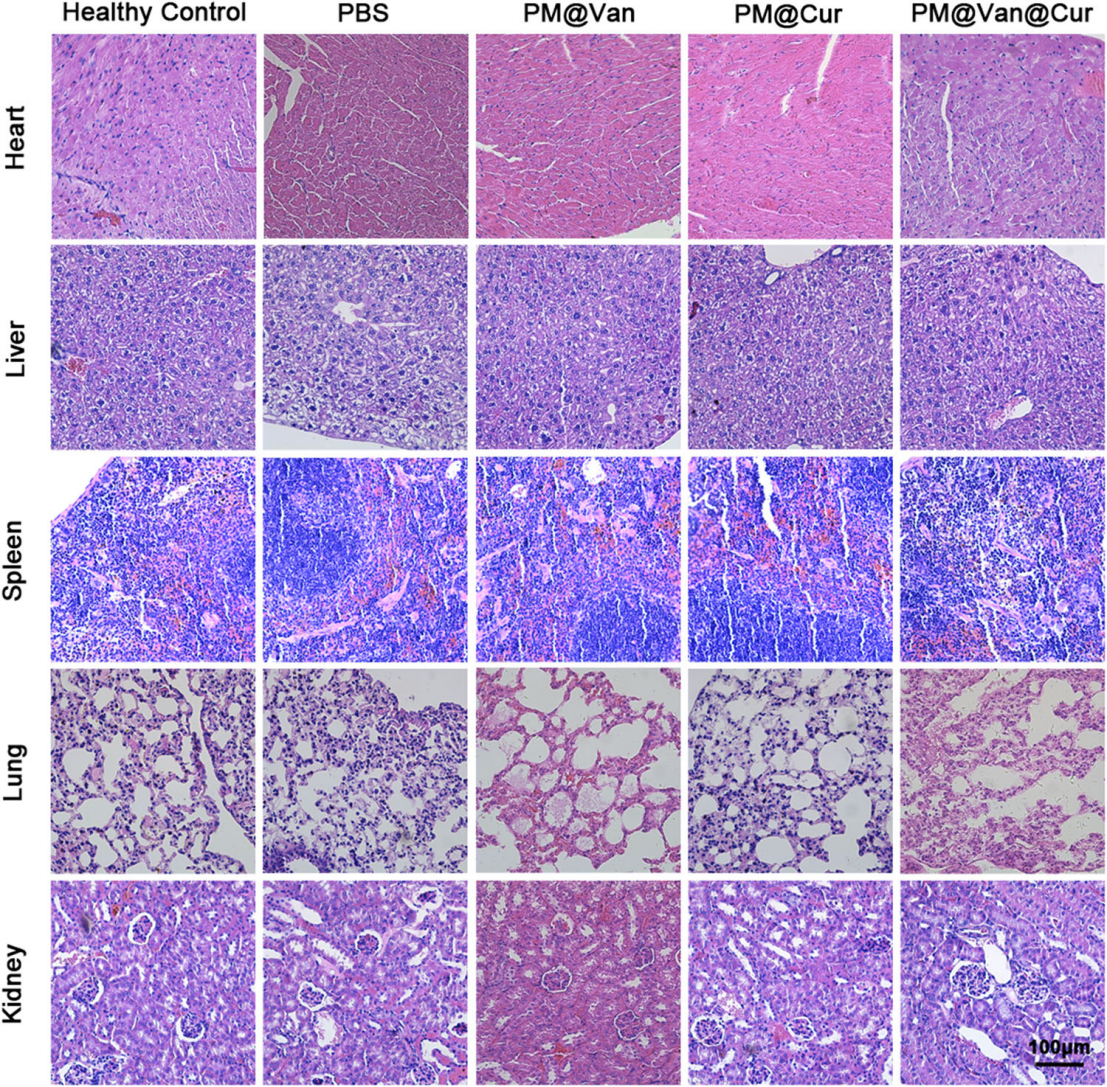
H&E staining images of main organs from mice after 5 days of different treatments

Because biosafety is a crucial concern when developing nanomedicine, we thus evaluated the biocompatibility of dynamic covalent polymeric antimicrobials through cytotoxicity, hemolysis, hematology, and histopathological analyses. From Figure 5A, the cell viabilities of 3T3 cells incubated with different micelles at concentrations from 0 to 500 μg ml^–1^ were all over 80%, evidencing their good cytocompatibility to normal cells. Moreover, after incubation of erythrocyte with different micelles, no obvious hemolysis was observed and all the erythrocytes maintained their regular biconcave disc morphology (Figure 5B,C). In addition, the main hematology indicators, including red blood cell, white blood cell, hemoglobin, platelet, mean corpuscular hemoglobin, mean corpuscular hemoglobin concentration, mean corpuscular volume, and hematocrit, were all in the normal range (Figure 5D), and no distinct histological damage in major organs could be found (Figure 6), which further proved that our dynamic covalent polymeric antimicrobials could be safely applied in vivo for treating bacterial infection disease.

## 3 CONCLUSION

In summary, we successfully developed a dynamic covalent polymeric antimicrobial using PBA‐functionalized micelle with co‐delivery of vancomycin and curcumin, which can serve as a new combination therapy for drug‐resistant bacterial infections. Benefiting from the reversible dynamic covalent interactions of PBA‐diols and π–π stacking interactions between two drugs, this antimicrobial could not only hold satisfying drug loading capability and formulation stability in normal physiological conditions but also achieve simultaneous access to the infection site and co‐release of the two drugs triggered by its acidic microenvironment, thereby exerting strong synergistic effect to kill the drug‐resistant bacteria without noticeable side effects. Moreover, this convenient strategy could also be adapted to other antibiotics with diol and aromatic structures, promising to be a universal platform for conquering drug‐resistant bacterial infection.

## 4 EXPERIMENTAL SECTION

Experimental details are provided in the Supporting Information.

## CONFLICT OF INTEREST

The authors declare no conflict of interest.

## ETHICS STATEMENT

All animal procedures were performed in accordance with the Guidelines for the Care and Use of Laboratory Animals of Peking Union Medical College and experiments were approved by the Animal Experiments and Ethics Review Committee of the Institute of Radiation Medicine, Chinese Academy of Medical Sciences (IRM‐DWLL‐2021058).

## Supporting information



Supporting Information

## Data Availability

All data related to this study are present in the article and in the Supporting Information. Any other data associated with this work are available from the corresponding authors upon request.
