# Supplementary material for: A dynamic covalent polymeric antimicrobial for conquering drug‐resistant bacterial infection
Source: Exploration (Beijing). 2022 May 23;2(5):20210145. doi: 10.1002/EXP.20210145 (PMC10191036; doi:10.1002/EXP.20210145)
Supplement: Supplementary file 1 — Supporting Information [file EXP2-2-20210145-s001.docx]

Supporting Information

**A Dynamic Covalent Polymeric Antimicrobial for Conquering Drug-Resistant Bacterial Infection**

*Fan Huang^#^, Xiaoyao Cai^#^, Xiaoxue Hou, Yumin Zhang, Jinjian Liu, Lijun Yang*, Yong Liu* and Jianfeng Liu**

F. Huang, X. Cai, X. Hou, Y. Zhang, J. Liu, L. Yang, Prof. J. Liu.

Key Laboratory of Radiopharmacokinetics for Innovative Drugs, Chinese Academy of Medical Sciences, and Institute of Radiation Medicine, Chinese Academy of Medical Sciences & Peking Union Medical College, Tianjin 300192, P.R. China

E-mail: yanglijun@irm-cams.ac.cn; liujianfeng@irm-cams.ac.cn

Prof. Y. Liu.

Engineering Research Center of Clinical Functional Materials and Diagnosis & Treatment Devices of Zhejiang Province, Wenzhou Institute, University of Chinese Academy of Sciences, Oujiang Laboratory (Zhejiang Lab for Regenerative Medicine, Vision and Brain Health), Wenzhou, Zhejiang 325001, P.R. China

E-mail: y.liu@ucas.ac.cn

EXPERIMENTAL SECTION

**Materials**

Vancomycin hydrochloride was purchased from Sigama-Aldrich Co. Ltd. Curcumin and Alizarin red S (ARS) were purchased from J&K chemical Co. Ltd. Hematoxylin-eosin (H&E) staining kit and Gram staining kit were purchased from Solarbio. Calcein AM/PI double stain kit was purchased from Yeasen biotech Co. Ltd. Mice IL-1β, IL-6, and TNF-α and IL-10 ELISA kits were purchased from Biolegend. Dimethyl formamide (DMF) and Dimethyl sulfoxide (DMSO) were bought from Tianjin Concord Co. Ltd. Ultrapure Milli-Q water (resistance >18 MΩ cm^−1^) was used to make all aqueous solutions.

**Synthesis of the polymers**

The experiment details for the synthesis and characterization of the block polymers, namely PEG-PLys(Z) and PEG-*b*-P(Lys-*co*-LysPBA), have been previously reported by our group.^[1]^

**Preparation of drug-loaded polymeric micelles**

To prepare curcumin-loaded polymeric micelles (PM@Cur), 2.5 mg PEG-Plys(z) and 0.15 mg curcumin powder were mixed in 1 mL anhydrous DMF, and the solution was dropwise added into 9 mL of ultrapure water with vigorous stirring. The PM@Cur was formed immediately, and the resulting solutions were stirred for another 4 h at room temperature to stabilize the micelles. Finally, the solutions were dialyzed against deionized water for 3 days to completely remove the DMF, and the PM@Cur was obtained.

For preparation of vancomycin-loaded polymeric micelles (PM@Van), 2.5 mg PEG-*b*-P(Lys-*co*-LysPBA) was dissolved in 9 mL ultrapure water. 400 μL of vancomycin aqueous solution (2 mg/mL) was prepared and dropwise added into the polymer solution with vigorous stirring, followed by the same procedure as described above.

As for polymeric micelles co-loaded vancomycin and curcumin (PM@Van@Cur), 2.5 mg PEG-*b*-P(Lys-*co*-LysPBA) was dissolved in 9 mL ultrapure water. 0.8 mg vancomycin and 0.15 mg curcumin were dissolved in 1 mL anhydrous DMF and added dropwise into the polymer solution under magnetic stirring. The PM@Van@Cur was formed immediately, and the resulting solutions were stirred for another 4 h at room temperature for stabilization. Then the solutions were dialyzed against deionized water for 3 days to completely remove the DMF and the PM@Van@Cur was obtained.

**Characterization studies**

PM@Van, PM@Cur and PM@Van@Cur were characterized by UV-visible absorption spectra using an UV-visible spectrophotometer (UV-2550, Shimadzu, Japan) in the spectral range from 250 nm to 700 nm. A fluorescence spectrophotometer (Hitachi F-4600, Japan) was used to record fluorescence spectra. The sizes of PM@Van, PM@Cur and PM@Van@Cur were measured by a laser light scattering spectrometer (BI-200SM) equipped with a digital correlator (BI-9000AT) at 636 nm. Transmission electron microscopy (TEM) measurements were performed with a commercial Philips T20ST electron microscope at an acceleration voltage of 100 kV.

**Drug loading content and drug loading efficiency of vancomycin and curcumin**

For measurement of drug loading content (DLC) and drug loading efficiency (DLE), PM@Van, PM@Cur and PM@Van@Cur was diluted into 50-fold volume of DMF. And the content of vancomycin was detected by a UV-vis spectrophotometer (at 280 nm) with free vancomycin as standard. The content of curcumin was determined by a fluorescence spectrometer (excitation at 420 nm and emission at 495 nm) with free Curcumin as standard. DLC and DLE of vancomycin or curcumin were calculated as the following formulas:

DLC (wt %) = (weight of loaded drug/total weight of polymer and loaded drug) ×100 %

DLE (%) = (weight of loaded drug/total weight of drug in feed) × 100 %

**ARS fluorescence measurement**

For the confirmation of the existence of phenylboronate ester formed by phenylboronic acid and vancomycin, fluorogenic assay of alizarin red S (ARS) was utilized. 1 mL phosphate buffer (pH 7.4) including 0.025 mM ARS and 0.25 mg mL^-1^ PEG-*b*-P(Lys-*co*-LysPBA) was put into a 10mL cuvette. Subsequently, a 5 μL of 5 mg mL^-1^ vancomycin in PBS (pH 7.4) was added into the above prepared solution each time, with final concentration of vancomycin ranging from 0.05 to 0.30 mg mL^-1^. Finally, pH value of the mixture solution was adjusted to 5.0. Allowing the solution to react for 5 min at a room temperature for every step, the mixture solution was performed fluorescence detection with excitation wavelength of 468 nm and then emission spectra were collected from 450 nm to 750 nm. Both excitation and emission slit widths were set at 10 nm.

**Evaluation of stability of PM@Van@Cur *in vitro***

The stability of PM@Van@Cur under different pH conditions was evaluated by using DLS. The sample was prepared by filtering PM@Van@Cur solution through 0.45 μm millipore filter. Changes of the sample in relative scattering light intensity with time were recorded at pH 5.0 and 7.4 separately.

***In vitro* release of vancomycin and curcumin in PM@Van@Cur**

Release of vancomycin and curcumin in PM@Van@Cur *in vitro* were carried out under different pH (pH 5.0 and 7.4) conditions to further verify the pH responsiveness of the micelles. 1 mL PM@Van@Cur micelles solution was transferred to dialysis bag (MWCO 3500). Subsequently the dialysis bag was submerged into 20 mL of PBS solution at pH 5.0 or pH 7.4 and incubated under stirring (37 °C, 200 rpm). At regular intervals, 1 ml of dialysis fluid was taken out to determine the amount of vancomycin and curcumin, and then an equal volume of fresh PBS solution with same pH value was added in. The amount of released vancomycin was determined by a UV-vis spectrophotometer (at 280 nm) with free vancomycin as standard. The amount of released curcumin was determined by a fluorescence spectrometer (excitation at 420 nm and emission at 495 nm) with free curcumin as standard. The release experiments were conducted in triplicate and the results presented are the average data.

**Antimicrobial assay against vancomycin-resistant staphylococci *in vitro***

One colony of bioluminescent *S. aureus* Xen36 was harnessed to inoculate into 10 mL of tryptone soya broth (TSB) with 200 μg mL^-1^ kanamycin, aerobically incubated at 37 °C for 24 h. Then, 2 mL of above culture medium was transferred to 40 mL of TSB, followed by the aerobic incubation at 37 °C for another 16 h. Subsequently, the bacteria were harvested by centrifugation (5000 g, 5 min, 4 °C), then washed twice by PBS (pH 7.4) under vortex for 3 × 10 s to disperse the bacteria. Finally, the bacteria were resuspended in 10 mL TSB to a concentration of 3 × 10^8^ bacteria mL^-1^ determined by a Bürker-Türk counting chamber.

For the evaluation of the bactericidal activity of PM@Van, PM@Cur, PM@Van@Cur, 100 μL of each micelle suspension with different micelle concentrations (ranging from 4 to 500 μg mL^-1^) were added into 96-well plates. Then different micelle suspension was mixed with equal volume of *S. aureus* Xen36 suspension in TSB (bacterial concentration of 3 × 10^8^ bacteria mL^-1^). PBS mixed with the bacteria was used as control. After incubated aerobically at 37 °C for 0, 4, 8, 12 h, the bioluminescence of the samples was measured by a bio-optical imaging system (IVIS Lumina II Imaging System, Perkin Elmer) for assessment of the antimicrobial activity of each polymeric micelle. Bioluminescence images (image acquisition factors: 20 s exposure time, medium binning, 1 F/Stop, Open Emission Filter) were automatically corrected for background noise. Regions of interest (ROIs) were manually created for each well and average radiances over the ROIs were converted to photon fluxes (p/s) using Living Image software (Perkin Elmer).

500 μL of *S. aureus* Xen36 suspension in TSB (bacterial concentration of 3 × 10^8^ bacteria mL^-1^) was added into a 1.5 mL micro-centrifuge tube, mixed with 500 μL of PM@Van, PM@Cur or PM@Van@Cur, and aerobically incubated at 37 °C for 120 min. Removing the supernatant after centrifugation (3000rpm, 3min), the precipitate was repeatedly washed thrice using the assay buffer solution. Bacteria were resuspended by the assay buffer solution to a concentration of 1 × 10^8^ bacteria mL^-1^. Then, 200 μL of bacterial suspension was incubated with 100 μL stain solution (calcein acetoxymethyl ester (Calcein-AM) (20 μM) and propidium iodide (PI) (5 μM)) at 37°C for 15 min. Subsequently, fluorescent image of bacteria in each group was recorded using fluorescence microscope with excitation wavelength 490nm. PBS solution mixed with *S. aureus* Xen36 suspension was used as control group.

**Checkerboard assay**

To evaluate the synergism between vancomycin and curcumin against *S. aureus* Xen36, briefly, *S. aureus* Xen36 (10^5^ bacteria mL^-1^) were mixed with vancomycin (0-64 µg mL^-1^) and curcumin (0-1000 µg mL^-1^) according to the typical synergy checkerboard assay. After being cultured at 37 °C for 24 h, the optical density (OD) in each well was measured on a Molecular Devices SpectraMax M2 precision microplate reader. To quantify the interactions between vancomycin and curcumin, Fractional Inhibitory Concentration (FIC) index was calculated as follows:

FIC Index = FIC_A_ + FIC_B_ = Van/MIC_van_ + Cur/MIC_Cur_

where Van and Cur are the MIC of vancomycin and curcumin in combination (in a single well), respectively, and MIC_van_ and MIC_Cur_ are the MIC of vancomycin and curcumin. The FIC Index value is then used to categorize the interaction of the two antibiotics tested (FIC < 0.5 for synergy, > 4 for antagonism, 0.5-4 for additive or indifference).

***In vivo* fluorescence imaging of Cy5 labelled polymeric micelles**

To prepare Cy5 labelled polymeric antimicrobial (PM@Van@Cy5), 2.5 mg PEG-*b*-P(Lys-*co*-LysPBA) was dissolved in 9 mL ultrapure water. 0.8 mg vancomycin and 0.15 mg Cy5 were dissolved in 1 mL anhydrous DMF and added dropwise into the polymer solution under magnetic stirring. The PM@Van@Cy5 was formed immediately, and the resulting solutions were stirred for another 4 h at room temperature for stabilization. Then the solutions were dialyzed against deionized water for 2 days to completely remove the DMF and the PM@Van@Cy5 was obtained. For the preparation of Free Cy5 solution, 0.15 mg Cy5 was dissolved in 1mL DMF, and then the Cy5-DMF solution was added into 9 mL PBS to form Free Cy5 solution.

In order to establish the infected murine model, one subcutaneous infection site was set in each mouse by injecting a dose of 2 × 10^7^ bioluminescent *S. aureus* Xen36 in the right flank of mice to start infection. The infected mice were randomly divided into two groups of five each at the third day after infection, after which each group received i.v. injection (i)200 μL free Cy5 solution (ii) 200 μL PM@Van@Cy5. Free Cy5 was performed as control. Fluorescence imaging was carried out 0.5h, 1h, 4h, 8h, 24h after injection (Excitation filter 630nm, Emission filter 700 nm long pass). The quantification of the fluorescence was performed by Image J.

**Eradication of vancomycin-resistant staphylococcal infection *in vivo***

In order to establish the infected murine model, one subcutaneous infection site was set in each mouse by injecting a dose of 2 × 10^7^ bioluminescent *S. aureus* Xen36 in the right flank of mice to start infection. Bioluminescent intensity and area were detected by a bio-optical imaging system (IVIS, 45 s exposure time, medium binning, 1 F/Stop, Open Emission Filter). Regions of interest (ROIs) were manually created for each mouse and average radiances over the ROIs were converted to photon fluxes (p/s) using Living Image software (Perkin Elmer).

The infected mice were randomly divided into four groups of six each at the third day after infection, after which each group received treatment every other day through intravenous injection (i) 200 μL PBS (pH 7.4) (ii) 200 μL 500 μg mL^-1^ PM@Van suspension (iii) 200 μL 500 μg mL^-1^ PM@Cur suspension (iv) 200 μL 500 μg mL^-1^ PM@Van@Cur suspension. Bioluminescent imaging was carried out every day. Treatment was started at 2nd day after infection and continued for consecutive 5 days.

**Measurement of inflammatory cytokine levels**

The infected tissues of mice sacrificed in each group were collected at the end of the treatments. The tissues were mixed with PBS and ground on ice. Then it was transferred into a micro-centrifuge tube and centrifuged for 10 min at 3000 rpm at 4°C. The resulting supernatant was divided into aliquot and stored at -80°C for later analysis. After centrifuged for 10 min at 3000 rpm, serum was harvested from the blood and then stored at -80 °C. IL-1β, IL-6, IL-10 and TNF-α in the supernatants and serum were measured with the ELISA kits according to the manufacturer’s instructions.

**Intracellular protein leakage measured via a BCA protein assay.**

*S. aureus* Xen36 (2×10^7^ bacteria mL^-1^) were mixed with vancomycin (2 µg mL^-1^), curcumin (15 µg mL^-1^), and vancomycin + curcumin (2 µg mL^-1^ vancomycin + 15 µg mL^-1^ curcumin), respectively, in PBS. After being incubated for 4 h at 37°C, bacterial suspension was centrifuged at 5000 rpm for 10 min at 4 ℃. The intracellular protein leakage in the supernatants was measured using a BCA protein detection kit.

**Cytotoxicity assay**

NIH 3T3 cells were employed for the MTT assay. The cells were placed into sterile 96-well plates at a concentration of 8000 cells well^-1^, attaching for 24h at 37 °C in a 5% CO_2_ incubator. Then, PM@Van, PM@Cur or PM@Van@Cur of varying concentration from 8 to 500 μg mL^-1^ was added into each well. After incubation for 24 h, 20 μL of a 5 mg mL^-1^ MTT solution was added to each well, incubated at 37 °C for another 4 hours. Finally, the mixtures were removed and 150 μL of DMSO was added, followed by a slight shaking for 5 minutes. Absorbance of formazan was measured at 570 nm through a Varioskan Flash (Thermo Scientific Company, USA). Cells without micelles were used as the control.

**Hemolysis assay**

Rat erythrocytes were extracted from fresh blood through centrifuging (5000 rpm for 5 min), repeatedly washed with PBS (pH 7.4) for three times. For the hemolysis assay, 5% Erythrocytes were resuspended in PBS gently, after which 0.5 mL of micelle solution (PM@Van, PM@Cur or PM@Van@Cur) with a range of concentrations of 15, 31, 62, 125, 250 and 500 μg mL^-1^ were respectively mixed with an equal volume of 5% erythrocytes suspension, followed by incubation at 37 °C for 1 h. Subsequently, the suspensions were centrifuged at 5000 rpm for 5 min, followed by a step that 100 μL of the supernatant was transferred into a 96-well plate to determine the free hemoglobin concentration through measuring the absorbance at 540 nm, using a Varioskan Flash (Thermo Scientific Company, USA). Meanwhile, the precipitate was transferred to a microscope slide for the observation of erythrocyte morphology, using microscope (eyepiece 10×, oil lens 63×). 0.1% Triton X-100 capable of damaging the red blood cells causing hemolysis was employed as positive control and PBS was used as negative control. The percentage of hemolysis was calculated as follows:

Hemolysis percentage (%) = (absorbance of sample - absorbance of negative control)/(absorbance of positive control - absorbance of negative control) ×100

**Histological examination**

Immediately the treatments were finished at day 5, the infected tissues and other major organs (spleen, lung, heart, liver, and kidney) of mice sacrificed in each group were collected and fixed in 4% formaldehyde solution, after which tissues were paraffin-embedded and sliced into 5 μm specimens. Subsequently, the organs were stained with hematoxylin-eosin (H&E) staining for biocompatibility evaluation, aside from which, the infected tissues were dyed with hematoxylin-eosin (H&E) staining and Gram staining for histopathology evaluation, respectively.

**Blood routine examination**

BABL/c female mice (19~21 g each) were randomly assigned into four groups of five each, after which each group respectively received intravenous injection (i) 200 μL PBS (ii) 200 μL PM@Van suspension (iii) 200 μL PM@Cur suspension (iv) 200 μL PM@Van@Cur suspension. Blood of each group was collected from eye socket at one day and one week later. For blood analysis, the micellar effects on blood were assessed by blood routine examination, including white blood cell counts (WBC), red blood cell count (RBC), hemoglobin concentration (HGB), platelet count (PLT), erythrocyte mean corpuscular volume (MCV), hematocrit (HCT), erythrocyte mean corpuscular hemoglobin (MCH) and erythrocyte mean corpuscular hemoglobin concentrate (MCHC), using automated hematology analyzer.

**Statistical analysis**

Statistical analysis was carried out using IBM SPSS Statistics software. All data were shown as mean ± SD. Statistical significance was analyzed using one-way ANOVA (**P* < 0.05, ***P* < 0.01).

**SUPPLEMENTARY DATA**

**Table S1.** Drug loading capacity (DLC) and drug loading efficiency (DLE) of different micelles

| **Sample** | **DLC of vancomycin (%)** | **DLC of curcumin (%)** | **DLE of vancomycin (%)** | **DLE of curcumin (%)** |
| --- | --- | --- | --- | --- |
| PM@Van | 22.8 | － | 92.3 | － |
| PM@Cur | － | 5.1 | － | 89.6 |
| PM@Van@Cur | 21.4 | 5.0 | 85.1 | 87.7 |

**
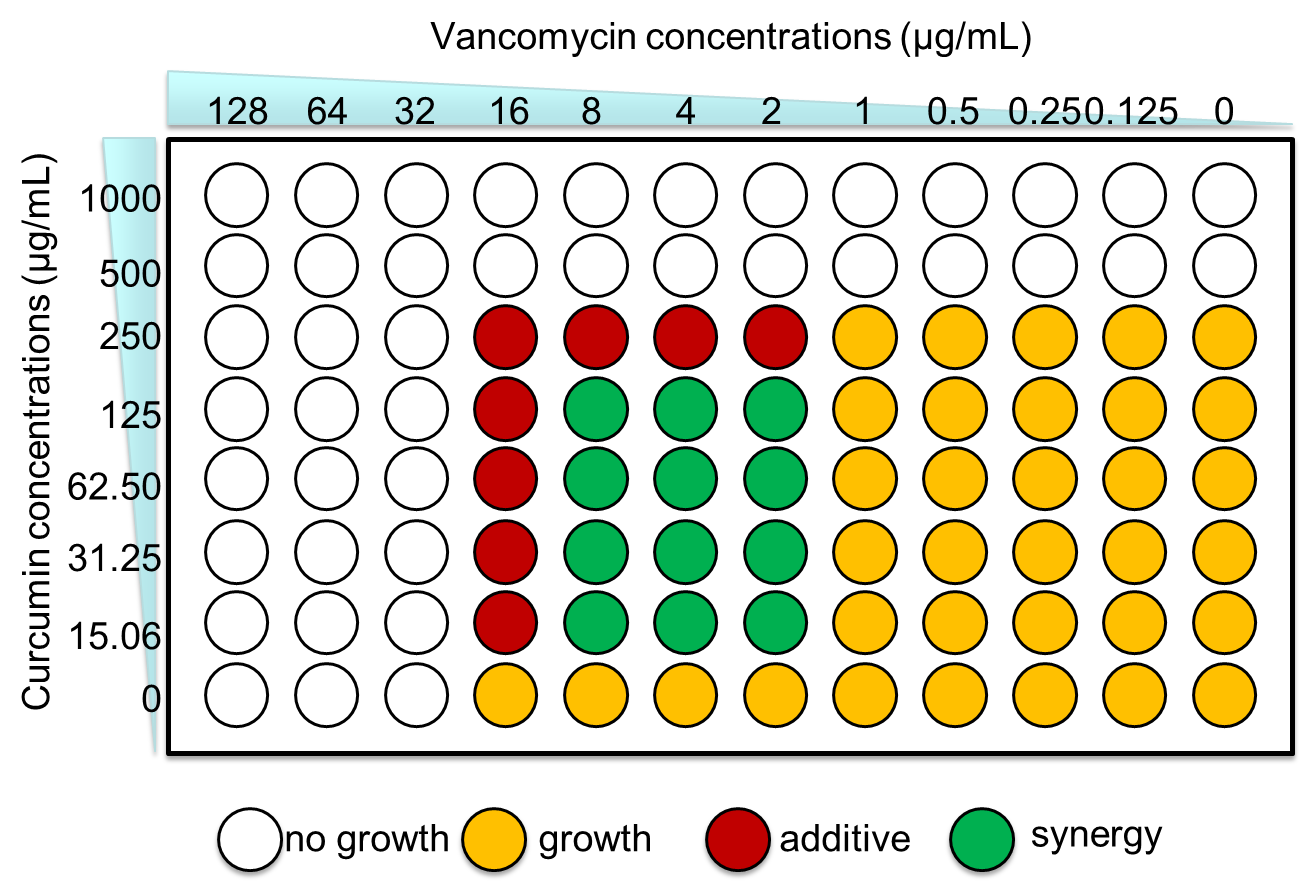
**

**Figure S1.** Checkerboard assay depicting the synergism between vancomycin and curcumin against *S. aureus* Xen36.

**Table S2.** MIC values and FIC of vancomycin combined with curcumin against the vancomycin-resistant *S. aureus* Xen36

|  | Antimicrobial susceptibility (MIC, μg mL^-1^) | | Antimicrobial combination  (MIC, μg mL^-1^) | | | | |
| --- | --- | --- | --- | --- | --- | --- | --- |
|  | vancomycin | curcumin | Van + Cur | | FIC | Potentiation | Interpretation |
| *S. aureus* Xen36 | 32 | 500 | 2 + 15.06 | 0.093 | | 16-fold | synergy |

Van: Vancomycin; Cur: Curcumin; MIC: Minimal inhibitory concentration; FIC: Fractional Inhibitory Concentration.


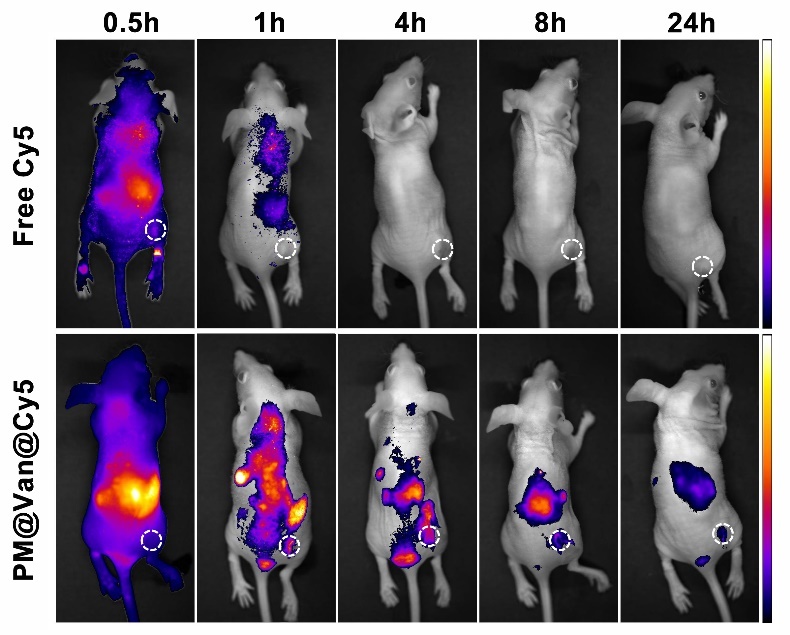


**Figure S2.** *In vivo* fluorescent images of infected mice post injection of free Cy5 and PM@Van@Cy5. Bacterial infection regions are marked by white circles.


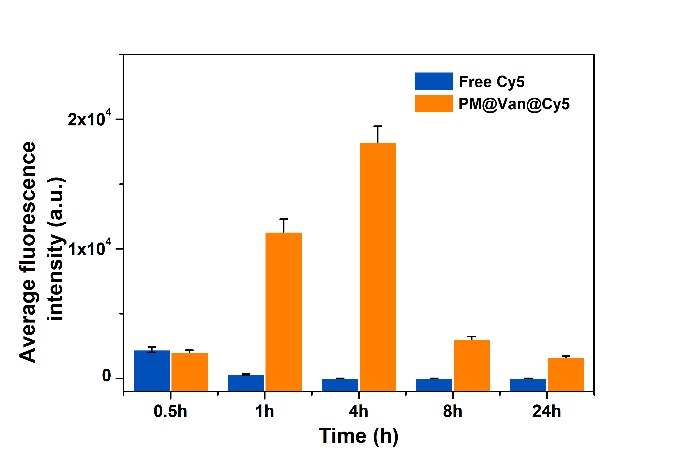


**Figure S3.** The average fluorescence intensities of the infected region post injection of free Cy5 and PM@Van@Cy5.


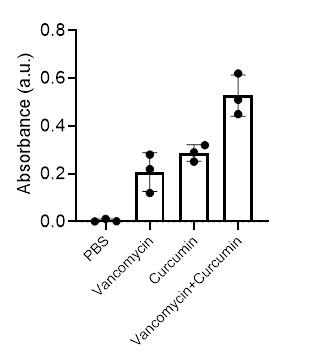


**Figure S4.** Intracellular protein leakage of S. aureus Xen36 after various treatments.

**References**

[1] T. Cheng, J. Liu, J. Ren, F. Huang, H. Ou, Y. Ding, Y. Zhang, R. Ma, Y. An, J. Liu, L. Shi, *Theranostics*. **2016**, 6, 1277.
